# Influences of Fucoxanthin on Alveolar Bone Resorption in Induced Periodontitis in Rat Molars

**DOI:** 10.3390/md14040070

**Published:** 2016-03-30

**Authors:** Oguz Kose, Taner Arabaci, Hatice Yemenoglu, Adem Kara, Seckin Ozkanlar, Sevki Kayis, Zeynep Yesil Duymus

**Affiliations:** 1 Department of Periodontology, Faculty of Dentistry, Recep Tayyip Erdogan University, Rize 53100, Turkey; htcymnglu@hotmail.com; 2 Department of Periodontology, Faculty of Dentistry, Ataturk University, Erzurum 25000, Turkey; t-arabaci@hotmail.com; 3 Department of Histology and Embryology, Faculty of Veterinary Medicine, Ataturk University, Erzurum 25000, Turkey; ademkara_36@hotmail.com; 4 Department of Biochemistry, Faculty of Veterinary Medicine, Ataturk University, Erzurum 25000, Turkey; seckin.ozkanlar@atauni.edu.tr; 5 Department of Aquaculture, Faculty of Fisheries Sciences, Recep Tayyip Erdogan University, Rize 53100, Turkey; aquasevki@msn.com; 6 Department of Prosthodontics, Faculty of Dentistry, Recep Tayyip Erdogan University, Rize 53100, Turkey; zeynep.yesilduymus@erdogan.edu.tr

**Keywords:** animal model, antioxidant, experimental periodontitis, fucoxanthin, oxidative stress

## Abstract

The aim of this study was to evaluate the effects of systemic fucoxanthin treatment on alveolar bone resorption in rats with periodontitis. Thirty rats were divided into control, experimental periodontitis (EP), and experimental periodontitis-fucoxanthin (EP-FUCO) groups. Periodontitis was induced by ligature for four weeks. After removal of the ligature, the rats in the EP-FUCO group were treated with a single dose of fucoxanthin (200 mg/kg bw) per day for 28 consecutive days. At the end of the study, all of the rats were euthanized and intracardiac blood and mandible tissue samples were obtained for biochemical, immunohistochemical, and histometric analyses. Fucoxanthin treatment resulted in a slight decrease in tumor necrosis factor-α, interleukin-1β, and interleukin-6 levels and a significant decrease in oxidative stress index. It was observed that fucoxanthin caused a significant reduction in receptor activator of nuclear factor kappa-β ligand (RANKL) levels and a statistically non-significant elevation in osteoprotegerin and bone-alkaline phosphatase levels. There were no significant differences in alveolar bone loss levels between the EP and EP-FUCO groups. This experimental study revealed that fucoxanthin provides a limited reduction in alveolar bone resorption in rats with periodontitis. One of the mechanisms underlying the mentioned limited effect might be related to the ability of fucoxanthin to inhibit oxidative stress-related RANKL-mediated osteoclastogenesis.

## 1. Introduction

Periodontitis is an infectious disease in which periodontopathogenic bacteria play an initial role; the course and severity is determined by the host response against the pathogen bacteria. Clinical findings include gingival hemorrhage, connective tissue destruction, pocket formation, and alveolar bone resorption [1,2].

Bone resorption results from osteoclast activity, which is controlled mainly by the receptor activator of the nuclear factor kappa-β (RANK)–RANK-ligand (RANKL)–osteoprotegerin (OPG) signaling pathway [3,4]. RANKL, a polypeptide that is bound to RANK on the cellular membranes of osteoclast precursors called RANKL receptors, induces these cells to convert to mature osteoclasts and promotes their activity. OPG, a glycoprotein that is produced by several cell types, including osteoblasts, fibroblasts, and endothelial and epithelial cells, inhibits osteoclastogenesis by inhibiting RANK/RANKL interaction [3,5].

The close relationship between RANKL level and elevated RANKL/OPG ratios and alveolar bone resorption in periodontitis is well known [3,6,7,8]. Pro-inflammatory mediators such as tumor necrosis factor-alpha (TNF-α), interleukin (IL)-1β, and IL-6 have been shown to up-regulate RANKL-mediated osteoclastogenesis [3,9], These mediators also increase the production and release of reactive oxygen species (ROS) [10]. Current studies have revealed that increased ROS production and oxidative stress play an important role in osteoclast formation through the upregulation of RANKL [11,12].

ROS are highly reactive, short-lived products of oxygen metabolism produced in biological systems. Oxidative stress, caused by changes in the dynamic balance between ROS production and antioxidant capacity, results in cells and tissues becoming more susceptible to oxidative damage [13]. Neutralization of the harmful effects caused by excessive ROS production and oxidative stress, including lipid peroxidation, oxidation of enzymes, and DNA and protein damage by antioxidant defense systems, is of vital importance in the maintenance of health [13,14]. Oxidative stress levels in body fluids or tissues can be determined by three methods: direct measurement of ROS, measurement of the final products of ROS, and determination of antioxidant levels. Malondialdehyde (MDA) is the final product that is used widely for assessing oxidative damage levels in lipid tissue, and 8-hydroxylguanosine is the final product used widely for assessing oxidative damage levels in DNA. The measurement of various enzymatic antioxidant levels, including superoxide dismutase, catalase, and glutathione peroxidase, is often preferred for assessing antioxidant status [15]. Erel [16,17] recently developed total oxidant status (TOS) and total antioxidant status (TAS) tests, as well as the oxidative stress index (OSI), for assessing the oxidant and antioxidant statuses of individuals. OSI is a proportional value between TOS and TAS, and it better defines oxidant/antioxidant imbalances.

The use of therapeutic agents with immunomodulatory and/or antioxidant properties is a popular approach for modulating hyperinflammatory host response in periodontal disease management [6,7,15,18,19,20,21]. Some current studies have reported the therapeutic effects of fucoxanthin (FUCO), a marine carotenoid, including antioxidant, anti-inflammatory, and anti-obesity effects [22,23]. The most important feature of FUCO that distinguishes it from the other carotenoids, such as beta-carotene and lutein, is its unique allenic carbon; this structural property accounts for its high antioxidant efficacy [22,24,25]. Current animal studies have reported that FUCO treatment significantly reduced ROS levels [26,27] and increased antioxidant enzyme levels and total antioxidant capacity [26]. FUCO has also been shown to inhibit TNF-α, IL-1β, and IL-6 levels significantly [28,29]. These effects of FUCO might comprise a useful therapeutic approach for various inflammatory diseases, including periodontitis.

In the current study, we hypothesized that FUCO could limit inflammation-mediated alveolar bone resorption through its antioxidant and immunomodulatory effects. As such, the aim of this study was to evaluate the effects of FUCO administration on alveolar bone resorption through biochemical, histochemical, and histometric analysis.

## 2. Results

### 2.1. Biochemical Results

#### 2.1.1. Serum TNF-α, IL-1β, and IL-6 Levels

The animals did not show obvious signs of drug toxicity. Serum TNF-α, IL-1β, and IL-6 levels were higher in the EP group compared to the control group (*p* < 0.05). FUCO administration provided a statistically insignificant reduction in the serum levels of those mediators (*p* > 0.05) (Table 1).

#### 2.1.2. Serum TOS, TAS, and OSI Levels

Serum TAS levels were lower (*p* < 0.05) and TOS levels and OSI were higher (*p* < 0.05) in the EP group compared to the control group. FUCO administration increased TAS levels (*p* < 0.05) and reduced TOS levels and OSI compared to the EP group (*p* < 0.05) (Table 1).

#### 2.1.3. Serum B-ALP Levels

The administration of FUCO resulted in a statistically non-significant increase in B-ALP level (*p* > 0.05), which was significantly lower in the EP group (*p* < 0.05) (Table 2).

### 2.2. Stereologic Results

**Figure 1:**
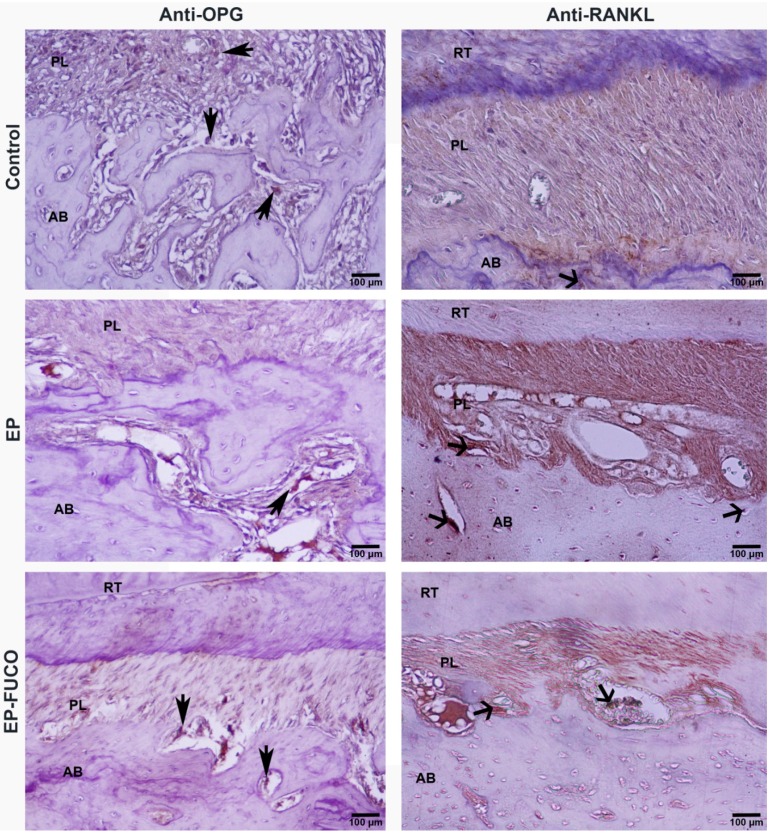
Illustration of immune-histochemical staining of mandibular sections of rats with anti-OPG and anti-RANKL for all groups, arrow head; OPG-positive cells (osteoblasts), open arrows; RANKL-positive cells (osteoclasts). AB: alveolar bone; PL: periodontal ligaments; RT: root; Streptavidin-peroxidase staining.

Figure 1 shows the immunohistochemical micrographs of the anti-RANKL and anti-OPG-stained sections. The numerical density values of the anti-RANKL-positive osteoclasts located in the resorption lacunae of the alveolar bone were higher in the EP group than in the control group (*p* < 0.05); however, FUCO reduced the anti-RANKL-positive osteoclasts significantly compared to the EP group (*p* < 0.05). The numerical density of the OPG-positive cells was significantly lower in the EP group compared to the control group (*p* < 0.05). FUCO administration resulted in a statistically non-significant increase in OPG-positive osteoblasts (*p* > 0.05) (Table 2).

### 2.3. Histometric Results (Alveolar Bone Loss)

**Figure 2:**
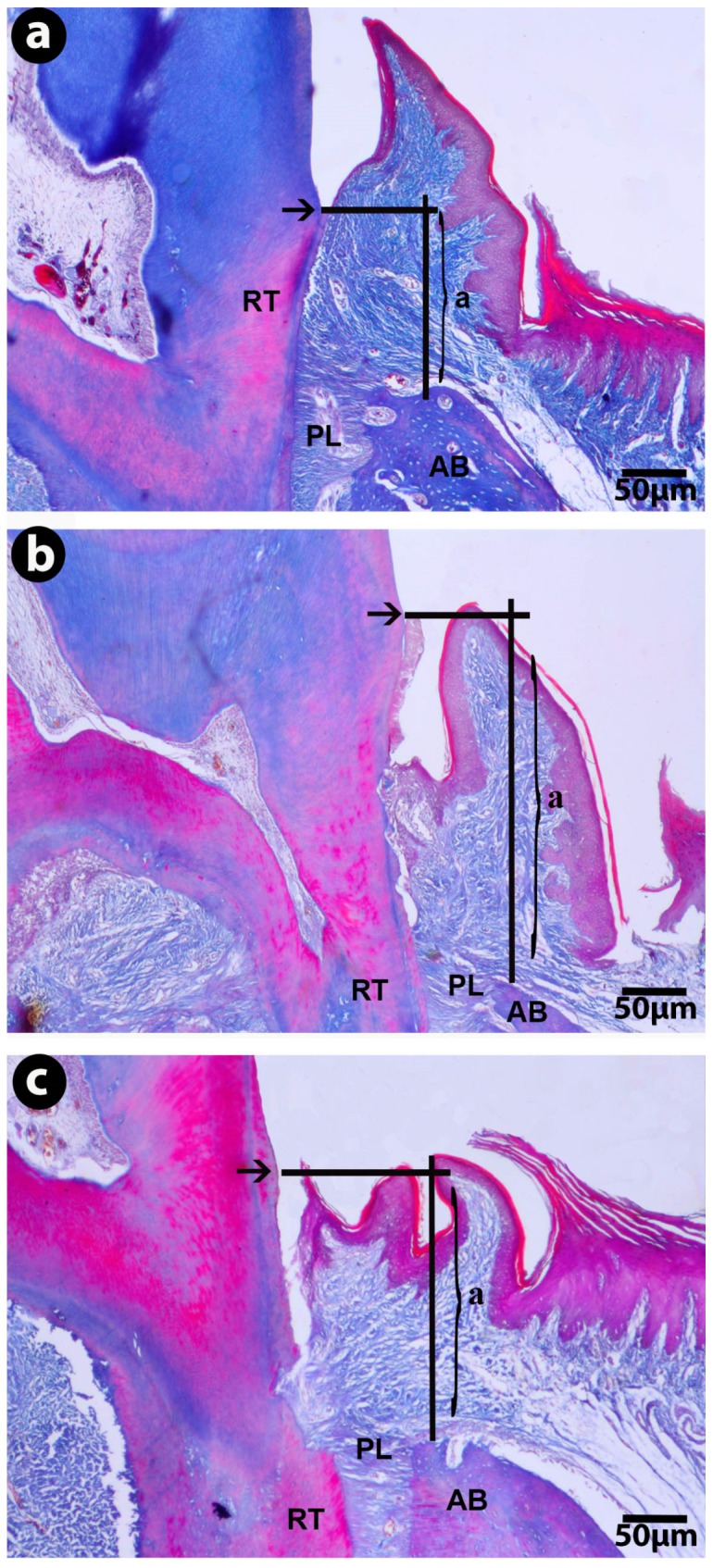
Micrographs of gingival mucosal tissues in the buccolingual sections of mandibular first molars showing alveolar bone loss levels, (**a**); control groups section, overview for measurement of cementoenamel junction-alveolar bone crest distance, (**b**); EP group section, (**c**); EP-FUCO groups section, arrow; cementoenamel junction; PL: periodontal ligaments; AB: alveolar bone; RT: root; Crossman modified triple staining.

The CEJ-BC distance was significantly higher in the EP group compared to the control group (*p* < 0.05). FUCO administration resulted in a statistically non-significant reduction in alveolar bone loss (*p* > 0.05) (Table 2, Figure 2).

## 3. Discussion

While a few studies [30,31] have evaluated the influence of various carotenoids on periodontal tissue destruction, to the best of our knowledge no studies are available in the literature investigating the influences of FUCO, a product whose therapeutic effects have been clearly shown, on periodontal tissue destruction. In the literature, several FUCO administration protocols in rats have been described [22,27,28,29]. Thirteen-week oral dosage studies previously showed that the critical FUCO dose at which toxicity and mutagenicity are not observed is 200 mg/kg bw [32,33]. Kadekaru *et al.* [34] reported that they found no toxicity after administering oral doses of FUCO (95% purity) to rats for 28 days. In the current study, the first report on this issue, 200 mg/kg bw of FUCO were administered intragastrically once a day for 28 days, as in previous studies [32,35].

The current experimental periodontitis study revealed that systemic FUCO treatment had a limited therapeutic effect on ligature-induced periodontitis-related alveolar bone resorption. The results of this study suggest that this limited effect might be related to the potent antioxidant effects of FUCO.

In this study, RANKL, OPG, and B-ALP activities were analyzed in order to evaluate alveolar bone resorption. The RANK-RANKL binding blockage capacity of OPG determines osteoclast maturation, activation, and resorption [3,4]. Pro-inflammatory cytokines released during host response and ROS are of vital importance in the production of RANKL and OPG by various cells [9,12,36]. The results of the current study, showing that RANKL activity increased in a statistically significantly manner [6,19,37] and that OPG activity decreased slightly [6,38] in periodontitis cases compared to controls, are consistent with those of the previous studies. The changes in RANKL and OPG that provoked resorption might be due to increased pro-inflammatory mediator (TNF-α, IL-1β, IL-6) levels and oxidative stress.

OSI may be a proper oxidative stress biomarker, as it is affected directly by oxidative and antioxidant status and clearly reflects final oxidative status [17]. Current studies have emphasized that OSI might be a practical method of assessing oxidative stress in periodontal diseases [14,15]. The results of the present study, showing that TOS levels and OSI are higher and TAS levels are lower in rats with periodontitis compared to controls, are consistent with those of previous studies [14,15,39]. It was observed in this study that systemic FUCO administration inhibited oxidative stress and supported antioxidant capacity. The significant reduction in OSI following treatment indicates that FUCO could limit oxidative damage in periodontal tissues. Nomura *et al.* [40] reported that because FUCO has six oxygen atoms, it might be more susceptible to free radicals, particularly under anoxic conditions. Therefore, the low oxygen content in the periodontal pocket might have contributed to the antioxidant effectiveness of FUCO in gingival tissues.

Many current studies have called attention to the potent antioxidant effects of FUCO, supporting our results [22,25] Zhang *et al.* [25] found that the superoxide radical-scavenging activity of FUCO and its stereoisomers is poorer than that of tocopherol (vitamin E), and that its hydroxyl radical-scavenging activity is more potent than that of tocopherol. They also reported that radical-scavenging activity is dose dependent. Recently, Tan and Hou [27] evaluated the anti-inflammatory effects of 0.2%, 0.4%, and 0.6% FUCO administration (intragastric, 28 days) in diet-induced obese rats, and they found that 0.6% FUCO, in particular, provided a significant reduction in serum MDA and polymorphonuclear cell infiltration (myeloperoxidase activity) levels in obese rats. The administration of 0.6% FUCO was also shown to provide a significant reduction in TNF-α and IL-1β levels. The authors evaluated the antioxidant effects of FUCO administration in undifferentiated PC12 cells and found that 0.4% and 0.6% FUCO reduced ROS production significantly and increased enzymatic antioxidant levels.

The results of the present study showed that FUCO administration did not have a significant effect on serum TNF-α, IL-1β, or IL-6 levels or local OPG activity, which increased significantly in rats with periodontitis. In addition, the histometric analysis findings indicated that FUCO did not have a significant effect on ligature-induced alveolar bone loss in the EP group. However, significant reductions in local RANKL activities (osteoclast density) were detected. The reduction in RANKL levels in the EP-FUCO group suggests that FUCO might limit RANKL-mediated osteoclastogenesis and alveolar bone resorption. Das *et al.* [41] reported that FUCO inhibited RANKL-mediated osteoclastogenesis and induced apoptosis in osteoclast-like cells. The findings of the current study suggest that the reduction in RANKL provided by FUCO is associated with FUCO’s potent antioxidant effectiveness rather than with its anti-inflammatory properties. The preponderance in antioxidant efficiency might be related to local environmental factors in the periodontal pocket, such as low oxygen content, which enhances the free radical susceptibility of FUCO. In support of this finding, studies have noted that FUCO acts as an antioxidant under anoxic conditions, whereas other carotenoids have practically no quenching abilities [24,40].

B-ALP, an enzyme produced by osteoblasts and is necessary for mineralization, is an important indicator of osteoblast activity and bone formation [42,43]. The results of this study showing that serum B-ALP levels decreased significantly in rats with periodontitis compared to controls are consistent with those of previous studies [19,20]. The significant reduction in B-ALP levels indicates that osteoblastic activity decreased in the periodontal area. The statistically insignificant elevation in B-ALP levels provided by systemic FUCO treatment in rats with periodontitis suggests that this therapeutic agent does not have a significant effect on osteoblasts in periodontitis tissues.

The small number of subjects and the inability of the biochemical and immunohistochemical methods used to provide comprehensive information regarding the immunomodulatory effects of FUCO are the two important limitations of this rat study. In addition, dose-response and time-course analyses were not performed. It is clear that new and comprehensive studies designed to reveal the effects of FUCO on periodontal tissue destruction and mechanisms of action are required.

In summary, this experimental periodontitis study revealed that the limited reduction in inflammation-mediated alveolar bone resorption provided by systemic FUCO administration might be associated with the antioxidant property of FUCO. Current studies [22,27] have highlighted the potent anti-obesity effects of FUCO. Since obesity is known to aggravate periodontal tissue destruction by provoking an inflammatory response [44], the potential effects of FUCO on obesity-mediated periodontal tissue destruction might be an interesting field of study.

## 4. Experimental Section

### 4.1. Extraction and Purification of FUCO

FUCO, supplied by BGG Biol. Tech. Co. (Beijing, China), was in the form of an oily liquid extracted with ethanol from *Laminaria japonica* and then purified. The purity of the FUCO used in this study was approximately 96%.

### 4.2. Induction of Periodontitis and FUCO Administration

Thirty male Sprague–Dawley rats (200–220 g) obtained from the Ataturk University Experimental Research and Application Center were used in this study. The experiments were performed according to the ethical principles approved by the Animal Ethics Committee of Ataturk University (2014-53). All of the animals were housed under standard laboratory conditions (relative humidity 58%, light period 6:00 a.m. to 7:00 p.m., 21 ± 2 °C) and fed standard rat pellets and tap water *ad libitum*. The rats were divided randomly into three groups of ten rats each. One of the groups was selected as the control group (Control), and was not exposed to any procedure. Periodontitis was induced in the remaining two groups (experimental periodontitis group (EP) and FUCO administrated group (EP-FUCO)) by placing 3-0 cotton ligatures around the cervix of the mandibular first molars for four weeks, in accordance with previous studies [19,20,21,37,45,46]. After removal of the ligatures, the rats in the EP and EP-FUCO groups were exposed to different treatment procedures for 28 consecutive days. The EP group was given a single intragastric dose of 1 ml saline per day, and the EP-FUCO group was treated with a single 200 mg/kg bw intragastric dose of FUCO per day. The FUCO dose selected was the highest, but not toxic, dose based on previous studies on rats [27,32,33,34,35]. After the drug administration procedures, the animals were anesthetized, and intracardiac blood samples were collected. The rats were then euthanized, and bilateral mandibular tissues were obtained for histometric and immunohistochemical analysis.

### 4.3. Blood Sampling and Biochemical Assays

Blood samples were centrifuged at 3200 *g* for 10 min within one hour after collection. The sera were stored at −80°C until they were analyzed.

#### 4.3.1. Serum TNF-α, IL-1β, and IL-6 Assays

Serum TNF-α, IL-1β, and IL-6 concentrations were measured using rat-specific enzyme-linked immunosorbent assay kits (Invitrogen, Carlsbad, CA, USA) according to the manufacturer’s instructions. The results are expressed as mean (pg/mL) ± standard deviation (SD).

#### 4.3.2. Measurement of Serum TOS and TAS Levels and Calculation of OSI

Serum TOS and TAS levels were measured using novel automated measurement methods and commercially available kits (Rel Assay Diagnostics, MEGA TIP, Gaziantep, Turkey), developed by Erel [16,17]. The results were expressed as micromolar hydrogen peroxide equivalent per liter (μmol H_2_O_2_ equivalent/gram protein) for TOS and millimolar Trolox equivalent per liter (mmol Trolox equivalent/gram protein) for TAS. The OSI, a practical indicator of oxidative stress, was calculated using the following formula [17]: OSI (arbitrary unit) = TOS (μmol H_2_O_2_ Eq/L)/TAS (μmol Trolox Eq/L) × 100.

#### 4.3.3. Measurement of Serum Bone Alkaline Phosphatase (B-ALP) Activity

B-ALP activities were determined using diagnostic kits (Roche Diagnostics, Mannheim, Germany); the results were expressed as U/L.

### 4.4. Histological and Immunohistochemical Analysis

#### 4.4.1. Histological Imaging and Measurements

The removed mandibular molar tissues were fixed in 10% neutral buffered formalin for 72 h. Subsequently, the tissues were decalcificated in 6% nitric acid solution for a week. The solution was refreshed daily and the tissue decalcification was controlled by a needle during the last three days. Following the completion of the decalcification process, the tissues were dehydrated in a graded alcohol series, embedded in paraffin wax, and sectioned into 5 μm thicknesses using a microtome (RM2125RT; Leica Instruments, Nubloch, Germany) along the molars along a buccolingual plane, for histological evaluation using Crossman modified Mallory’s triple staining. Eight slides were obtained for each animal (total of 80 slides in each group). The distance between the cementoenamel junction (CEJ) and the alveolar bone crest (BC) was measured using a trinocular light microscope (Kameram SLR, 1.4.1.0; Mikro Sistem Ltd., Istanbul, Turkey) attached to analyzing software (Figure 2a–c).

#### 4.4.2. Immunohistochemical Analysis and Calculation of RANKL- and OPG-Positive Cells

The eight alveolar bone sections from each rat were stained with anti-RANKL (dilution: 1/50; Abcam) (Santa Cruz Biotech., Santo Cruz, CA, USA) and anti-OPG (dilution: 1/50; Abcam) (Santa Cruz Biotech., Santo Cruz, CA, USA) according to the manufacturer’s protocols, using an Avidin Biotin Complex (ABC) staining system. Binding of the antibodies was visualized with a high-power light microscope (Eclipse i50; Nicon, Tokyo, Japan). In order to estimate the immunopositive cell count, staining intensity was measured using the stereologic optical fractionator method, as described in detail in our previous studies [19,20].

### 4.5. Statistical Analysis

Because all of the data presented a normal distribution and the coefficient variation was less than 20%, differences between the groups were tested by analysis of variance and Duncan’s test, using SPSS 17.0 (SPSS Inc., IBM Company, Chicago, LA, USA). All data were expressed as mean average ± SD; *p* < 0.05 was considered significant.

## Figures and Tables

**Table 1:** Comparison of biochemical results between groups.

Groups	TNF-α (pg/mL)	IL-1β (pg/mL)	IL-6 (pg/mL)	TOS (μmol H_2_O_2_ Equiv/L)	TAS (mmol Trolox Equiv/L)	OSI (Ratio)
Control	44.4 ± 8.6 ^a^	4.5 ± 2.1 ^a^	13.4 ± 4.6 ^a^	16.43 ± 5.43 ^a^	1.14 ± 0.33 ^a^	1.36 ± 0.41 ^a^
EP	61.1 ± 10.2 ^b^	7.9 ± 3.6 ^b^	22.2 ± 8.1 ^b^	23.87 ± 4.62 ^b^	0.61 ± 0.26 ^b^	3.11 ± 0.67 ^b^
EP-FUCO	58.8 ± 7.1 ^b^	7.5 ± 2.8 ^b^	19.8 ± 6.7 ^b^	18.50 ± 4.68 ^a^	0.86 ± 0.24 ^c^	1.66 ± 0.38 ^a^

The results are expressed as mean ± standard deviation. ^a–c^ The footnote letters in the same column indicate significant differences between groups. ANOVA and *post-hoc* Duncan tests were performed (*p <* 0.05).

**Table 2:** Comparison of serum B-ALP levels and stereological and histometric results between groups.

Groups	Serum B-ALP (U/L)	Anti-RANKL Positive Cells (*n*/µm^2^)	Anti-OPG Positive Cells (*n*/µm^2^)	CEJ-BC (µm)	
Control	124.86 ± 12.40 ^a^	0.0000374 ^a^	0.0000877 ^a^	L	149.65 ± 11.36 ^a^
				B	143.25 ± 13.68 ^x^
EP	98.86 ± 9.40 ^b^	0.0000866 ^b^	0.0000397 ^b^	L	372.34 ± 32.47 ^b^
				B	367.23 ± 43.58 ^y^
EP-FUCO	105.10 ± 12.32 ^b^	0.0000402 ^a^	0.0000411 ^b^	L	338.37 ± 16.85 ^b^
				B	336.71 ± 15.86 ^y^

L, lingual; B, buccal; CEJ-BC, cementoenamel junction-alveolar bone crest. Values are expressed as mean ± standard deviation, except anti-RANKL- and anti-OPG-positive cell numbers. ^a,b^ and ^x,y^ The footnote letters in the same column indicate significant differences between groups. Statistical analyses were performed using ANOVA and *post hoc* Duncan tests (*p <* 0.05).
